# An Aberrant Case of Pheochromocytoma in a Young Adult Presenting With Secondary Hypertension: A Case Report With Review of the Literature

**DOI:** 10.7759/cureus.44891

**Published:** 2023-09-08

**Authors:** Deepali Trimukhe, Avinash Dhok, Suresh Phatak, Prashant Onkar

**Affiliations:** 1 Radiodiagnosis, NKP Salve Institute of Medical Sciences and Research Centre (NKPSIMS & RC) and Lata Mangeshkar Hospital (LMH), Nagpur, IND

**Keywords:** catecholamine-secreting tumour, neuroendocrine tumour, young hypertensive, light bulb appearance, adrenal paraganglioma, pheochromocytoma

## Abstract

Pheochromocytoma (PCC) is a rare neuroendocrine catecholamine-secreting tumour of the adrenal gland. It originates from the chromaffin cells found within the adrenal medulla or the extra-adrenal paraganglia. We present a case report of a 24-year-old female who presented with hypertension, headache, palpitations, chest pain and blurry vision. On ultrasound evaluation, a right suprarenal mass was noted, which was further evaluated using contrast-enhanced computed tomography (CT). Based on our imaging findings, the patient was diagnosed with a case of right-sided pheochromocytoma. The patient was operated on, and our diagnosis was confirmed with histopathological examination.

## Introduction

Pheochromocytoma (PCC) is a rare neuroendocrine catecholamine-secreting tumour of the adrenal gland. It grows from the chromaffin cells of the adrenal medulla or extra-adrenal paraganglia. PCC occurs in 0.1%-0.6% of hypertensive patients. The mean age of presentation varies, with a female-to-male ratio of 1.4:1 [1].

Approximately 40% of PCC cases are hereditary in origin. The most common syndromes associated with familial PCC are familial multiple endocrine neoplasia (MEN2A) or MEN2B, neurofibromatosis (NF1) and von Hippel-Lindau syndrome (VHL2) [2].

The clinical presentation of PCC shows a wide spectrum of symptoms ranging from asymptomatic presentation to cardiac arrest. The diagnosis of PCC is dependent on the identification and characterisation of a suprarenal mass on imaging with clinical, biochemical and histopathological confirmation [2]. The classical triad of secondary hypertension (caused by high catecholamine-secreting PCC), sweating and tachycardia has a 90% specificity for the presence of PCC, occurring in approximately 10%-36% of patients with PCC [1].

## Case presentation

Patient information

A 24-year-old female presented with chief complaints of headache, palpitation, chest pain and blurry vision for the past two months. The patient also developed hypertension and was prescribed medication by her general physician.

Clinical findings

On examination, vital parameters indicated blood pressure of 180/100 mmHg, heart rate of 104 bpm and respiratory rate of 24 breaths/minute. The patient was conscious and well-oriented. Haemoglobin, total leukocyte count, liver function test, kidney function test and D-dimer were normal.

Assessment on imaging

**Figure 1:**
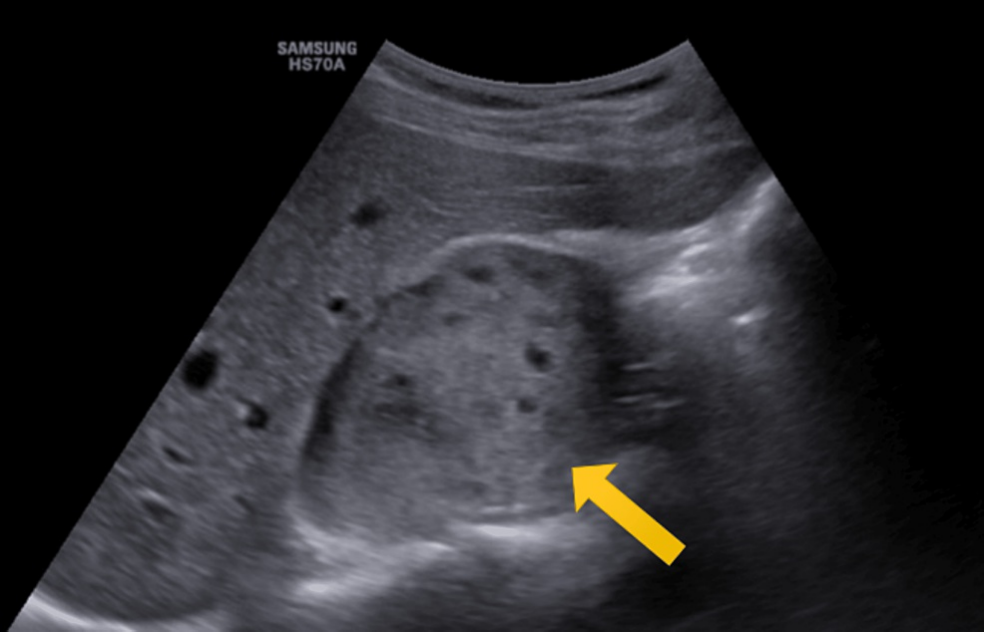
On B-mode ultrasound, an iso-hyperechoic mass in the right suprarenal region with multiple anechoic areas is shown (yellow arrow).

The right kidney was small in size, measuring 70 × 30 mm. An iso-hyperechoic well-defined solid mass sized 60 × 40 × 47 mm (transverse (T) × anteroposterior (AP) × craniocaudal (CC)) in the right suprarenal region was present. No calcification was present within the mass. Multiple small anechoic areas were also present within the mass, suggestive of necrotic changes (Figure 1).

**Figure 2:**
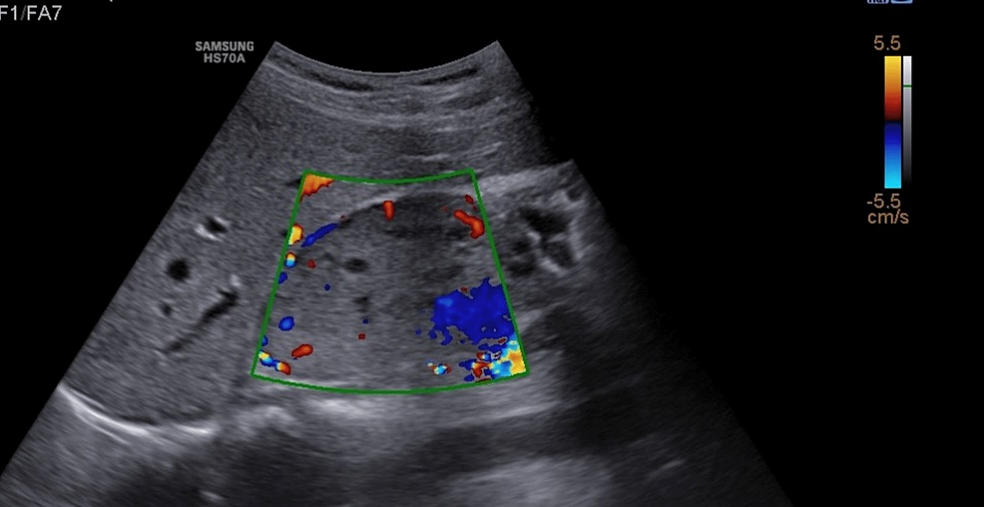
On colour Doppler, moderate peripheral and central vascularity is demonstrated.

On colour Doppler, the mass showed moderate peripheral and central vascularity (Figure 2).

The rest of the abdominal organs were normal.

**Figure 3:**
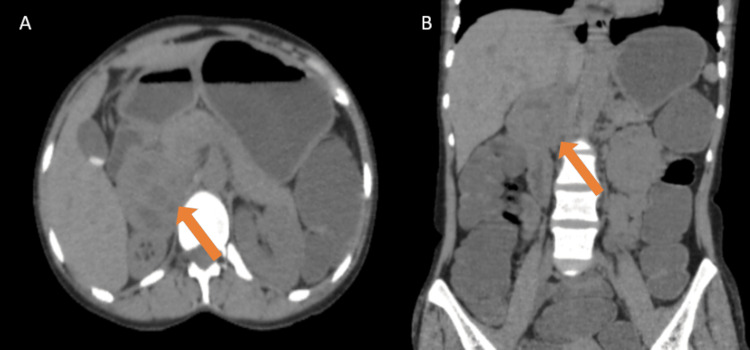
Plain axial (A) and coronal (B) CT images show an iso-hypodense lesion in the right adrenal region having smooth margins (orange arrows). CT: computed tomography

A well-defined soft tissue attenuation (+12 to +15 HU) lesion of approximately 53 × 47 × 56 mm (T × AP × CC) was noted involving the right suprarenal gland. Multiple hypodense areas were noted within this lesion (Figure 3).

**Figure 4:**
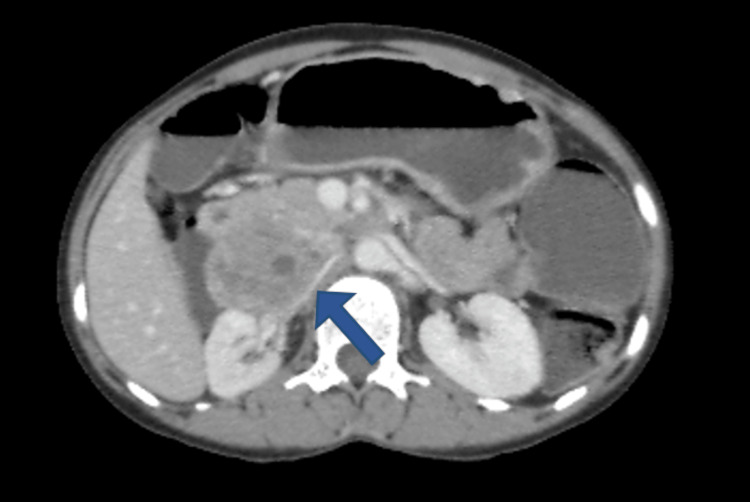
The lesion showed heterogenous enhancement with multiple non-enhancing areas within, representing necrosis, and the right renal artery was pushed posteriorly throughout its entire course by this lesion. However, it showed normal contrast enhancement (blue arrow).

In the post-contrast study, the lesion showed heterogenous enhancement with multiple non-enhancing areas within, representing necrosis. The right renal artery was pushed posteriorly throughout its entire course by this lesion; however, it showed normal contrast enhancement (Figure 4).

Based on the above imaging features, a diagnosis of pheochromocytoma was made.

This was followed by 24-hour urine vanillylmandelic acid (VMA), which was surprisingly within normal limits. The patient was advised to take phenoxybenzamine (an alpha-blocker) for 14 days prior to the excision of the lesion. Subsequently, the patient underwent a wide excision of the right adrenal lesion, along with a right adrenalectomy.

Histopathology

**Figure 5:**
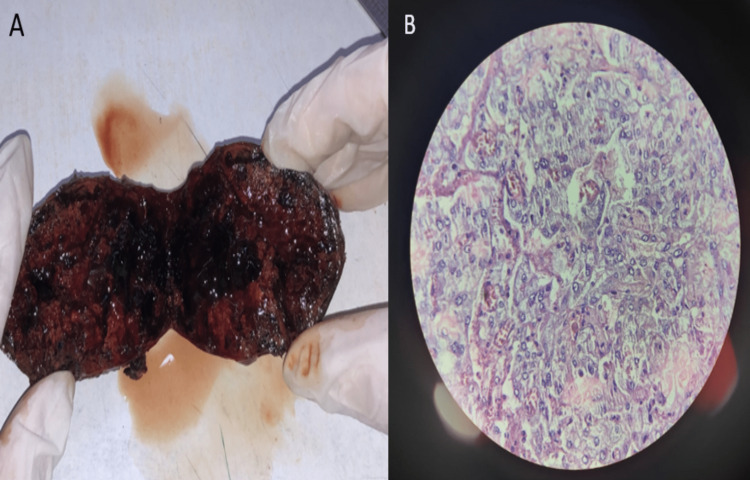
(A) The cut surface of the right suprarenal mass shows black and haemorrhagic areas. (B) On microscopy, the cut section of the mass shows the sheets and nests of tumour cells with scanty intervening stroma, consistent with the diagnosis of pheochromocytoma.

On the gross cut specimen, black haemorrhagic areas were noted. On microscopy, sheets and nests of tumour cells with scanty intervening stroma were noted. The tumour cells were large and polygonal with round to oval vesicular nuclei and clumped chromatin nucleoli, along with granular and eosinophilic cytoplasm. These findings confirm the histopathological diagnosis of pheochromocytoma (Figure 5).

Follow-up and outcome of intervention

The postoperative period proceeded without complications, and the patient made a satisfactory recovery. During the follow-up after two months, the patient’s blood pressure level was measured at 120/80 mmHg, which falls within the normal range. Additionally, other symptoms such as blurred vision, headache, palpitations and chest pain have also subsided.

## Discussion

PCC develops from chromaffin cells and secretes the excess catecholamines. Extra-adrenal PCC, also known as paragangliomas, arises from the paraganglionic sympathetic and parasympathetic nervous system. It tends to occur at any region extending from the base of the skull to the urinary bladder along the sympathetic and parasympathetic paraganglia. The organ of Zuckerkandl is a common location of extra-adrenal paragangliomas [3].

PCC appearance on ultrasound and computed tomography is variable, with solid to mixed solid-cystic appearance [1]. Tumours are round or oval in shape with similar attenuation to the surrounding soft tissue structure [4].

On post-contrast CT evaluation, it shows uniform or heterogenous enhancement and, mostly, enhancement of solid components. Washout of contrast usually overlaps with either benign lesions or malignant lesions; hence, they are difficult to differentiate on contrast evaluation alone. On magnetic resonance imaging (MRI) evaluation, it was hypointense on T1-weighted images (T1WI) and hyperintense on T2-weighted images (T2WI), which is the classical “light bulb appearance.” On post-contrast evaluation, it commonly enhances avidly. Metaiodobenzylguanidine (MIBG) scan is used in clinically suspected PCC for localisation and confirmation, as well as excluding metastasis. The sensitivity of MIBG is not high, but the specificity is 100%. Scintigraphy performed with somatostatin receptor analogues, such as octreotide, can be another alternative for the localisation of PCC. In clinical practice, 18F-fluoro-2-deoxy-d-glucose (FDG) positron emission tomography (PET) is a widely used modality in diagnosing PCC, which shows increased uptake on PET. However, this is seen in cases of adrenal metastases as well [5].

Management

The primary treatment for tumours remains surgical removal. Performing a needle biopsy in cases of clinically suspected pheochromocytomas is discouraged due to the potential risk of increased catecholamine hypersecretion. Preoperative management for pheochromocytoma involves alpha-blockers for 14 days prior to surgery to reduce complications. Beta-blockers can be used for tachycardia control, but only after alpha-blockers, and laparoscopic adrenalectomy is the preferred surgical approach [2].

## Conclusions

Pheochromocytoma is a rare catecholamine-producing tumour of the adrenal gland. In young hypertensives, PCC should be a suspected diagnosis. Numerous imaging modalities are used in evaluating pheochromocytoma, but ultrasound and CT are excellent modalities for early diagnosis, which also helps in optimum management.
